# Quantitative analysis of lung microwave ablation zone volume and shape

**DOI:** 10.1186/s41747-026-00721-2

**Published:** 2026-05-28

**Authors:** Robert S. Salkin, Arvind B. Dev, Erica S. Alexander, Amgad Moussa, Vlasios Sotirchos, Constantinos T. Sofocleous, Gilbert Maroun, Eslam W. Youssef, Mario Ghosn, E. Nadia Petre, Stephen B. Solomon, Krishna Nand Keshavamurthy, Etay Ziv

**Affiliations:** Department of Radiology, Memorial Sloan-Kettering Cancer Center, New York, NY USA

**Keywords:** Ablation techniques, Lung, Microwaves

## Abstract

**Objective:**

We quantified volume and shape variability in lung microwave ablation (LMWA) zones, comparing them with expected ablation zones, exploring the correlation with tissue contraction.

**Materials and methods:**

After Institutional Review Board approval, we retrospectively included patients who underwent LMWA between January 2015 and January 2019. Exclusion criteria were ablations with multiple burns/probes, overlapping ablation zones, or indistinguishable background lung parenchyma. Ablation zones were oriented along an applicator-centric coordinate system. We used Pyradiomics to generate volumes and the Euler characteristic transform for three-dimensional shape space analysis. Wilcoxon paired signed-rank tests compared the expected *versus* the actual ablation zone. Tissue contraction was quantified using paired anatomical landmarks before and after computed tomography scans.

**Results:**

We included 111 ablations in 72 patients (31 male, 41 female; median age 59). Median ablation power was 65 watts (range 20‒65), median ablation time 5 min (range 1‒10). Total energy correlated with volume and width (*p* = 0.007, *p* = 0.003, respectively). Ablation volume did not differ from vendor predictions (*p* = 0.452), whereas length (*p* < 0.001) and maximum width (*p* < 0.001) were greater than predicted. Ablation shapes were more elongated (*p* < 0.001), less spherical (*p* < 0.001), asymmetric (wider in back than in front, *p* = 0.007), and diverged from expected ellipsoids. There was no correlation between tissue contraction and volume, power, or time.

**Conclusion:**

The provided vendor model offers a reasonable estimate of mean ablation volume, but wide variability in volume and shape necessitates a more bespoke method of ablation zone prediction. Tissue contraction did not correlate with ablation zone variability.

**Relevance statement:**

High variability in actual ablation volume, shape, length, width, and position along the needle and divergence from ellipsoids necessitates the development of improved ablation zone prediction models.

**Key Points:**

- While median ablation volumes do not differ from vendor predictions, ablation length (*p* < 0.001) and maximum width (*p* < 0.001) are greater than vendor predictions, and volumes are variable.
- Ablation shapes diverge from ellipsoids, are more elongated (*p* < 0.001), less spherical (*p* < 0.001), and asymmetric (*p* = 0.007).
- Tissue contraction does not account for the volume variability of ablation zones.

**Graphical Abstract:**

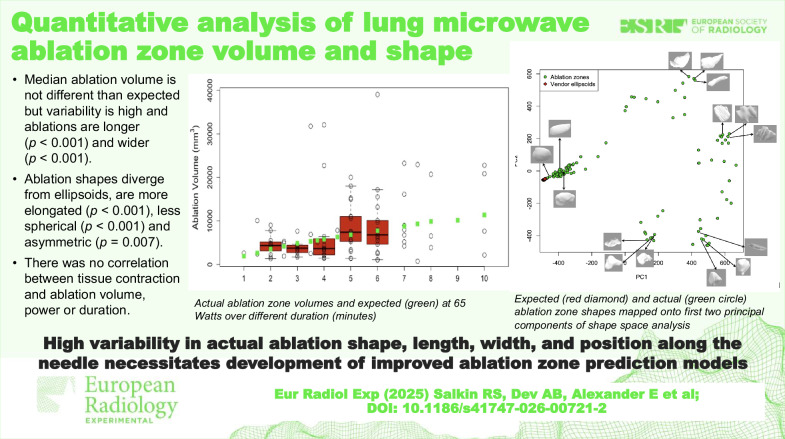

## Backgorund

Lung microwave ablation (LMWA) is a well-established procedure that can be used to treat primary or secondary cancers in the lung. It is minimally invasive, performed in the outpatient setting, and preserves pulmonary function, making it an attractive alternative to surgical resection or stereotactic body radiation therapy for patients with comorbidities or multiple tumors [1, 2]. Like surgical resection and stereotactic body radiation therapy, achieving adequate treatment margins during LMWA is essential to ensure complete tumor destruction and reduce local recurrence [3]. A significant challenge for LMWA is to achieve adequate margins [4, 5].

During the procedure, the operator selects treatment parameters to achieve an expected ablation zone size that will yield adequate margins. This is based on a vendor-provided table of expected ablation zone sizes corresponding to various power and durations of ablations. However, these are limited since: (a) they are based on *ex vivo* animal data; (b) they only provide expected values rather than information about variability or ranges; and (c) they do not account for patient-specific local anatomical factors that influence ablation zone size and shape. The first and third issues have been well-documented, including a study demonstrating the relationship between tumor location and the extent of the ablation zone [6] More recently, an animal model was used to quantify the variability of LMWA in an effort to capture *in vivo* data [7] Some authors have also suggested that the volume variability observed in LMWA is due to tissue contraction [8], which is the result of ablation zone involution and cicatrization over time [9].

In this study, a quantitative analyses of ablation zone volumes and three-dimensional (3D) shape was performed, along with comparison with the vendor’s expected values, and evaluation of ablation zone variability associated with tissue contraction.

## Methods

### Patient selection

**Fig. 1:**
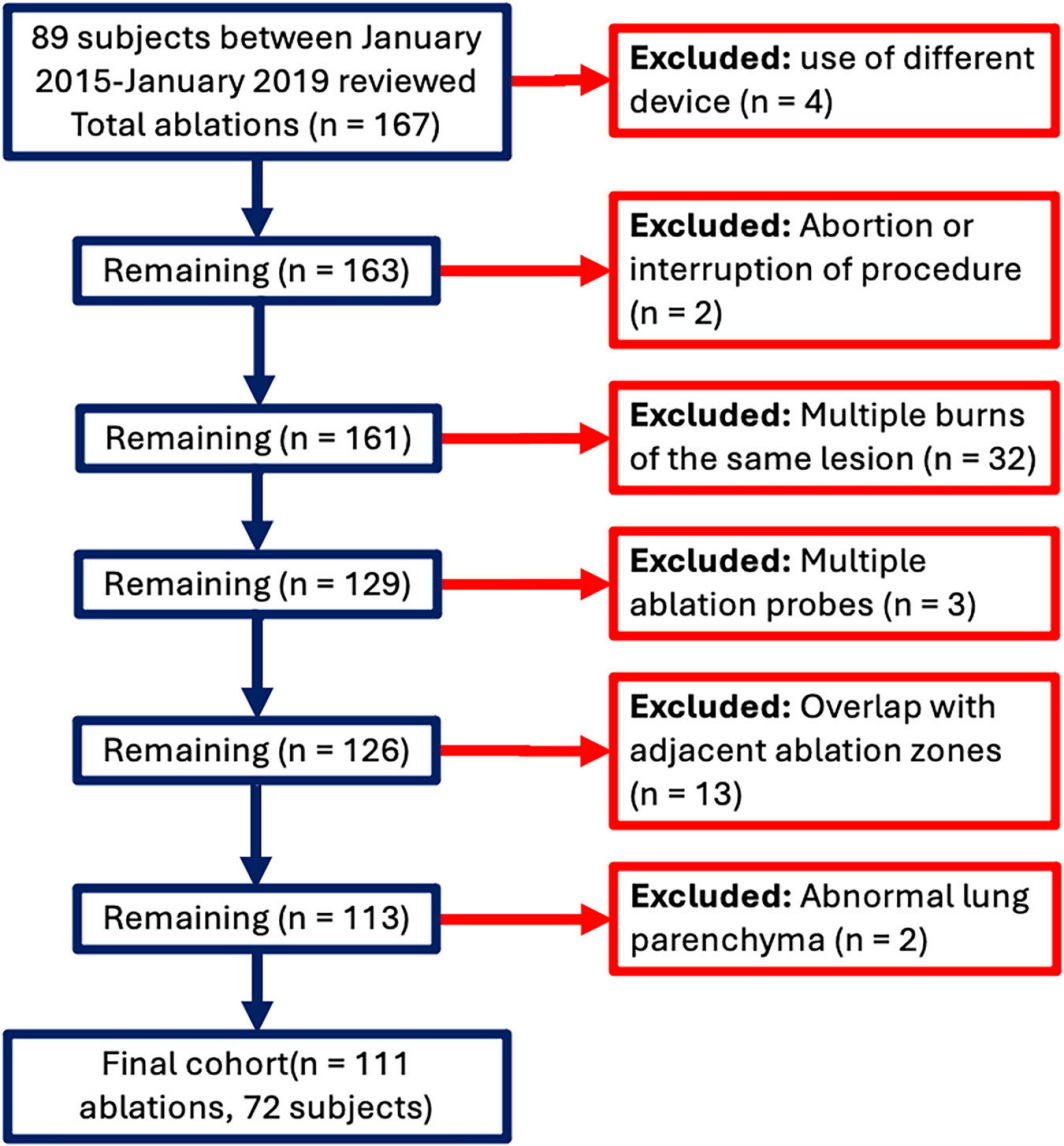
Flowchart of study cohort

Consecutive patients who underwent LMWA between January 2015 and January 2019 were included in this Institutional Review Board-approved single-institution retrospective study. This study is reported as per the STROBE guideline [10]. As the primary motivation was to quantify volume and shape variability in ablation zones and relate to the vendor-predicted chart of ablation zones, the cohort was limited to ablations that were single-probe and single-burn. Our dataset contains a final cohort of 111 ablations in 72 unique patients who underwent LWMAs using the 17-gauge Neuwave PR probe from Ethicon Endo-Surgery. Exclusion criteria included scans with abnormal background lung parenchyma (ablation zone not differentiable from background), multiple ablations at the same site, ablations with more than one probe at the same site, ablations performed with a non-Neuwave PR probe or other modality, or if two or more adjacent sites undergoing ablation at the same time resulted in overlapping ablation zones (Fig. 1).

### LMWA

The study follows standard lung thermal ablation techniques, terminology, and reporting guidelines [11]. All patients were evaluated in the IR clinic, and the decision to perform LMWA was made in conjunction with members of the thoracic disease management team. All subjects underwent a pre-procedure chest computed tomography (CT) scan (1.25-mm diagnostic slices). CT guidance was used for intraoperative applicator positioning and monitoring of LMWA. Fellowship-trained interventional radiologists performed all ablations. Ablation parameters were recorded, including modality, applicator used, number of applicators, applicator repositioning, and power and duration of each ablation. LMWA was performed under general anesthesia with conventional tidal ventilation. Choice of ablation wattage and time was at the operator’s discretion, with guidance from the vendor’s predicted ablation zone size. Adequate coverage assessment was based on the immediate post-procedure imaging appearance of the surrounding ground glass as per guidelines. All patients underwent standard-of-care post-procedure chest CT (1.25 mm) approximately four weeks post LMWA.

### Ablation zone segmentation

Ablation zone assessment was performed at the first follow-up CT. Segmentations were performed independently by two trainees and a board-certified interventional radiologist with over 10 years of experience, all blinded to the power and duration of the ablation. Discrepancies were resolved by consensus. Segmentations were performed using 3D Slicer (v5.2.1) [12]. Initially, a contour was manually drawn on the boundary of the ablation zone on each axial image in the lung window. Subsequent edits were performed on coronal and sagittal views.

### Vendor model

The vendor’s user interface software (v3.1.0) provides dimensions of expected ablation ellipsoids for different powers and durations at 5 watts and 1-min increments, respectively. Linear interpolation was performed to define expected ablation zone dimensions for power and duration between the specifications (35 watts to 65 watts and 1–10 min).

### Applicator-centric coordinate system (ACCS)

An ACCS [13]—a 3D coordinate grid oriented along the ablation applicator and centered at the applicator tip—was defined with the purpose of enabling comparison of ablation zones at different orientations across patients. The ACCS grid was set to 64 × 64 × 64 mm^3^ with a sampling of 1 mm along all three dimensions.

### Tissue contraction

Tissue contraction of the ablation zones was quantified by selecting common anatomical landmarks that were identifiable in both the pre-procedure and one-month post-procedure CT scans, such as blood vessel bifurcation points. A range of 3–10 landmarks close to the ablation zone was chosen for each pair of images. Pairwise Euclidean distances between all anatomical landmarks were then computed in both scans. Tissue contraction was defined as the mean change in distance between pre- and post-procedure scans.

### 3D shape space analysis

Topological data analysis was employed to encode information about the shape of a complex dataset [14, 15]. Specifically, the Euler characteristic transform, which does not require any mapping between shapes [16, 17], was applied to the ablation zone segmentations using the demeter Python package [18]. Briefly, each ablation zone was transformed to its dual cubical complex, and an Euler characteristic transform was applied over multiple directions (*n* = 256) and thresholds (*n* = 64). Each ablation zone was thus encoded as a 1 × 16,384 vector. A kernel principal component analysis (using an ANOVA kernel) was then applied for data visualization of the full dataset. Vendor-predicted ellipsoids were subsequently projected onto the space.

### Statistics

Volume and simple shape statistics (sphericity, elongation, surface-to-volume ratio) determined from ablation zone segmentations were calculated using PyRadiomics [19]. The length of the ablation zone was measured along the applicator’s orientation in the ACCS. In order to compare the ablation zones' short axis with a vendor ellipsoid short axis, we needed to define a short axis width and area for the ablation zones. To compute the ablation maximum width and ablation area, a mean diameter and area were computed at each 1 mm slice perpendicular to the ACCS long axis. The maximum mean diameter and maximum area over all the slices for each ablation were then defined as the ablation maximum width and ablation area, respectively (Supplementary Fig. S1). Results did not substantively change with the median or maximum of diameters used. A Kolmogorov-Smirnov test was used to quantify the normality of the ablation zone shape statistics across the whole cohort, and the Shapiro–Wilk normality test was used for different ablation subsets. Correlations between energy (power × time) and other covariates, as well as tissue contraction and other covariates, were calculated using a partial correlation coefficient to account for patients with multiple ablations [20]. Volume and shape statistics were compared with the corresponding vendor predicted model using the paired Wilcoxon sign ranked test with clustering to account for patients with multiple ablations, using clusrank package in R and the Rosner–Glynn–Lee method [21]. Reported significance was based on *p*-values adjusted for multiplicity. Similarly, clustered pairwise Wilcoxon (using clusrank and Rosner–Glynn–Lee method) was used to assess length, width, slice area, and volume differences between the front and back portions of ablation zones. A two-sample Mann–Whitney test was used to assess the significance of the difference in landmark distances for tissue contraction. Tumor characteristics (solid, subsolid, and cavitary), location (central/peripheral), adjacency to pleura, tumor volume, and applicator orientation to lung (tangent, normal, and in between) were recorded. Correlation between these and ablation zone volume was calculated on ablation zone subsets (same power and duration). To determine the extent to which covariates accounted for the discrepancies between observed ablation volume and tip to edge and the vendor predicted, a linear mixed-effects model was fit with discrepancy (log observed/vendor) as the outcome, tissue contraction (mm), time to follow-up (days), ablation energy, and tumor volume as fixed effects, and patient identity as random effect.

### Study subjects or cohorts overlap

Some study subjects or cohorts have been previously reported. A portion of this cohort was used in the development of the ablation zone prediction model, which is the focus of the study by Keshavamurthy et al [22]. The current study cohort is also included in the studies by Cooke et al [23] and by Geevarghese et al [24], which summarized outcomes after lung ablation in the entire cohort. While there is overlap in the cohorts, the current manuscript is a study of the quantitative volumetric and shape variation in ablation zones and their relation with the expected ablation zone volume and shape, and tissue contraction. None of these variables was measured or analyzed in the other papers. Moreover, there is no prediction modeling in this study and no discussion of recurrence or survival outcomes in this study.

## Results

There were 111 LMWA included in this cohort (Fig. 1), composed of 72 unique subjects (31 male, 41 female) with a median age of 59 years (range 18–85 years). Tumor histologies and origin are included in Table 1. 92% (66/72) of the patients had ablations of metastases to the lung. 64% (46/72) of patients had ablations of colorectal metastases. 86% (95/111) of nodules were solid, and 7% (8/111) were subsolid, and 7% (8/111) were cavitary. The mean nodule volume was 420 (±390) mm^3^. There were 23/111 (21%) nodules that were central, 47/111 (42%) that were within 5 mm of a pleural surface, 27/111 (24%) that were sandwiched between 2 or more vessels, and 45/111 (41%) that were within 5 mm of a vessel > 3 mm. The median ablation power was 65 watts (range 20–65 watts), and the median ablation duration was 5 min (range 1–10 min). The median time between the ablation procedure and post-procedure follow-up scan was 30 days (interquartile range [IQR] = 11 days). The four most common ablation parameter settings (power, duration) accounted for 54/111 (54%) of ablations and included: 65 watts, 5 min (18/111, 16.2%); 65 watts, 6 min (17/111, 15.3%); 65 watts, 4 min (14/111, 12.6%); and 65 watts 2 min (11/111, 9.9%).

**Table 1:** Patient and treatment variables

Ablations	111
Subjects	72
Age (years)	59 (18–85)
Sex	31 male, 41 female
Time to follow up (IQR)	30 days (11 days)
Histology	
Colorectal	46
Lung	10
Gynecological	
Uterine leiomyosarcoma	4
Cervix	1
Endometrial adenocarcinoma	1
Sarcoma	
Inferior vena cava	1
Chondrosarcoma	1
Osteosarcoma	1
Eye (adenoid cystic)	1
Genitourinary	
Prostate	1
Bladder	1
Hepatocellular Carcinoma	1
Esophageal	1
Pancreas	1
Wattage, W (range)	65 (20–65)
Ablation time, min (range)	5 (1–10)
Nodule characteristics	
Solid	95
Subsolid	8
Cavitary	8
Largest vessel within 5 mm	
< 2 mm	35
2–3 mm	31
> 3 mm	45
Within 5 mm of the pleural surface	47
Nodule between 2 or more vessels	27
Nodule volume (standard deviation)	420 (390) mm^3^
Location	
Central	23
Peripheral	88

*IQR* Interquartile range

### Volume and shape statistics

**Fig. 2:**
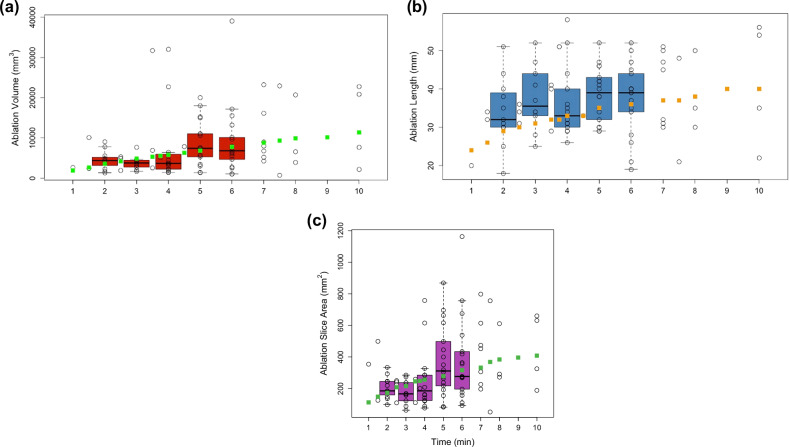
Data from the cohort for ablations at 65 watts at different durations for (**a**) volumes, (**b**) lengths, and (**c**) slice areas. Box plots show IQR for the five most common ablation times (2‒6 min). Green squares show vendor-predicted values. Ablation lengths are longer than vendor-predicted (*p* < 0.001)

Total energy was correlated with ablation volume (*p* = 0.007) and width (*p* = 0.003), but not length. The median ablation zone volume was 5.3 cm^3^ (IQR = 2.9–9.0 cm^3^), and the median volume of the vendor-predicted model was 6.2 cm^3^ (IQR = 4.8‒7.8 cm^3^). Figure 2 shows actual and vendor-predicted volumes, lengths, and areas across all 65-watt ablations. The median ablation length was 35 mm (IQR = 30‒44 mm), and the median ablation max width was 21.2 mm (IQR = 16.9–28.8 mm), Table 2. The ablation length was longer than the vendor’s length (*p* < 0.001), and the ablation maximum width was wider than the vendor’s width (*p* < 0.001). Ablation volume was not significantly different between vendors. The distance between the needle tip and the ablation zone’s front edge (median 6 mm, IQR = 4‒8 mm) was longer than the vendor-predicted distance (median 3 mm, IQR = 3‒4 mm, *p* < 0.001). Tumor characteristics (solid, subsolid, or cavitary), adjacent vessel size, nodule location, adjacency to pleura, and location between vessels were not associated with volume. Tumor size was correlated with ablation zone volume (*p* = 0.001).

**Table 2:** Ablation shape statistics

Ablation zone statistic (*n* = 111)	Median	Vendor-predicted median	Rank statistic	95% Confidence interval	Adjusted *p*-value
Volume	5.3 cm^3^	6.2 cm^3^	0.11	-0.17 to 0.39	0.45
Length	35 mm	33 mm	0.63	0.27–0.98	< 0.001
Max width	21.2 mm	18.6 mm	1.16	0.71–1.60	< 0.001
Tip to edge	6 mm	3 mm	2.30	1.54–3.06	< 0.001
Elongation	1.6	1.4	1.53	0.93–2.13	< 0.001
Sphericity	0.65	0.9	-6.00	-7.75 to -4.24	< 0.001
Surface to volume	0.42	0.3	3.10	2.20–4.00	< 0.001

Analysis of the sample distributions of ablation volumes for the four most common parameters (65 watts and 2, 4, 5, and 6 min, Table 3) was performed. The distribution skewness was positive for all power and time settings, while 65 watts, 6 min, and 65 watts, 4 min, had a skewness greater than one. All four subsets of ablation volumes had a kurtosis value greater than one. Ablation volume distributions are thus right-skewed and heavy-tailed.

**Table 3:** Ablation volume, length, maximum width, and tip-to-edge distributions for the four most common ablation parameters

65 Watts, 6 min					
	Median [IQR]	Mean absolute deviation	Skewness	Kurtosis	Vendor
Volume	6.8 [5.5] cm^3^	5.8 cm^3^	2.26	8.24	7.8 cm^3^
Length	39.0 [10] mm	7.3 mm	-0.48	2.45	36.0 mm
Max width	23.4 [9.2] mm	6.2 mm	0.44	2.87	19.75 mm
Tip to edge	5.0 [5] mm	2.8 mm	0.35	1.88	4.0 mm
**65 Watts, 5 min**					
	**Median [IQR]**	**Mean absolute deviation**	**Skewness**	**Kurtosis**	**Vendor**
Volume	7.4 [5.6] cm^3^	4.5 cm^3^	0.60	2.40	6.8 cm^3^
Length	39.0 [10.2] mm	5.8 mm	0.11	1.99	35.0 mm
Max width	24.47 [9.4] mm	6.1 mm/	0.04	2.32	18.5 mm
Tip to edge	7.0 [4.8] mm	3.0 mm	0.55	2.49	4.0 mm
**65 Watts, 4 min**					
	**Median [IQR]**	**Mean absolute deviation**	**Skewness**	**Kurtosis**	**Vendor**
Volume	3.6 [3.4] cm^3^	5.8 cm^3^	2.09	5.89	5.8 cm^3^
Length	33.0 [8.8] mm	7.0 mm	1.23	3.44	33.0 mm
Max width	18.7 [6.9] mm	5.5 mm	1.20	3.49	17.6 mm
Tip to edge	6.0 [2.8] mm	2.0 mm	0.63	3.09	3.0 mm
**65 Watts, 2 min**					
	**Median [IQR]**	**Mean absolute deviation**	**Skewness**	**Kurtosis**	**Vendor**
Volume	4.3 [2.0] cm^3^	1.7 cm^3^	0.49	2.54	3.5 cm^3^
Length	32.0 [9.0] mm	6.9 mm	0.17	2.72	29.0 mm
Max width	19.3 [3.5] mm	2.8 mm	0.05	2.89	14.77 mm
Tip to edge	6.0 [2] mm	1.4 mm	-0.25	2.20	3.0 mm

**Fig. 3:**
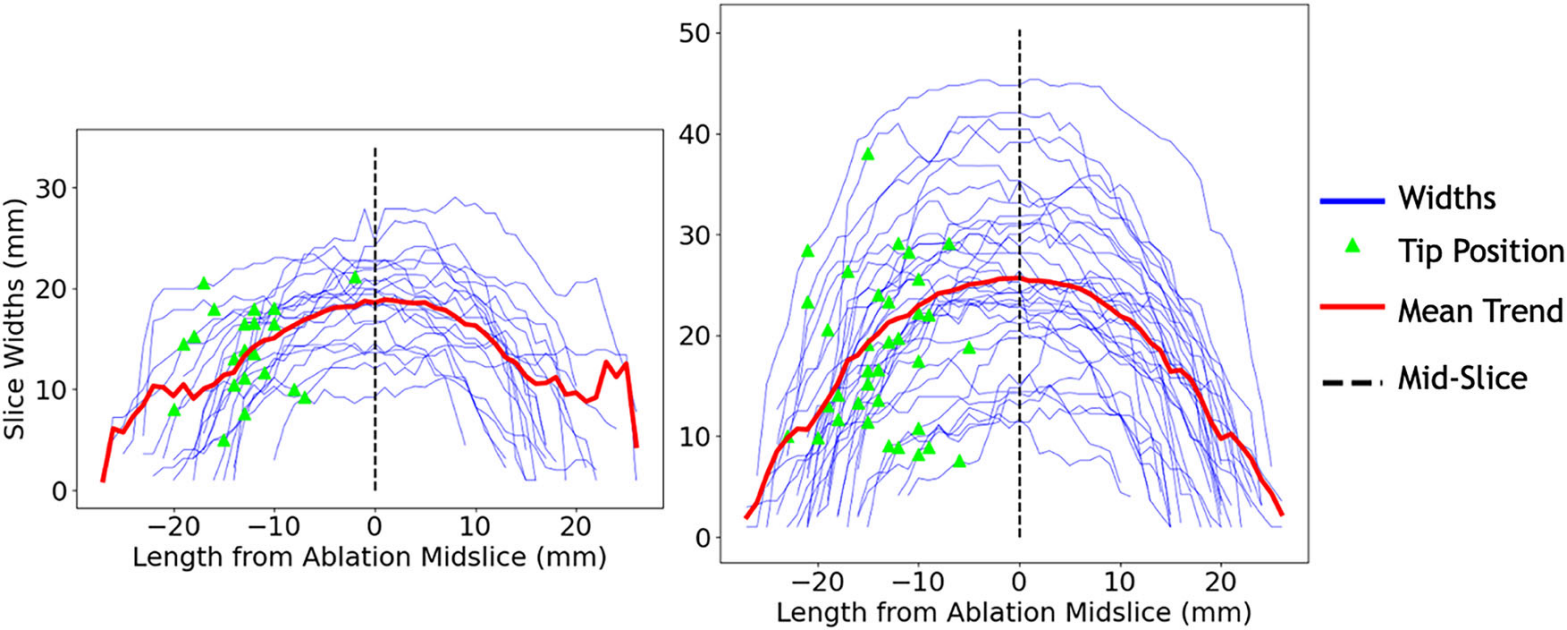
Ablation zone maximum widths for all 65-watt ablations at 2 or 3 min (**Left**) and 5 or 6 min (**Right**). Each line represents a single ablation, 0 is the ablation mid-slice, red line is the median. The green triangle represents the position of the needle tip along the x-axis for the corresponding ablation. Pairwise comparison of front *versus* back maximum widths shows asymmetry with narrower front *versus* back (*p* = 0.007)

The actual ablation zones were more elongated (*p* < 0.001), less spherical (*p* < 0.001), and had greater surface-to-volume ratios (*p* < 0.001) compared with the vendor-predicted ablations (Table 2). Energy correlated with elongation (*p* = 0.010) and surface-to-volume ratio (*p* = 0.006) but not sphericity. The ablation zones were found to be asymmetric: the width was smaller in the front half, towards the tip (median 16.1 mm, IQR = 10.6‒23.2) compared with the back half, away from tip (median 16.8 mm, IQR = 11.3‒23.1 mm, *p* = 0.007, Fig. 3).

### Tissue contraction

**Fig. 4:**
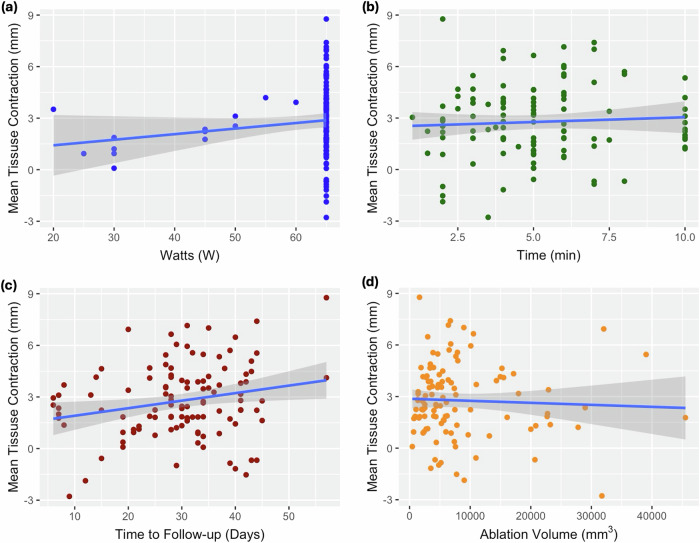
Correlations between mean tissue contraction after ablation and (**a**) power, (**b**) duration, (**c**) time to follow-up, and (**d**) ablation zone volume at first follow-up

The median tissue contraction between pre- and post-procedure follow-up CTs was 2.5 mm (range: -2.8 to 8.8 mm), with a mean distance of 0.9 mm between post-follow-up and pre-procedure. The tissue contraction was found to be normally distributed) for 90% (100/111) of ablations. Eight percent of ablations (9/111) had a mean tissue expansion between the pre-and post-procedure follow-up CTs. 75% (83/111) of ablations in the cohort had a significant difference in the median landmark distance between the post-procedure follow-up and pre-procedure scans. There was no observed correlation between tissue contraction and ablation zone volume (*r* = -0.05), ablation power (*r* = 0.16) or ablation time (*r* = 0.08), but there was a correlation (*r* = 0.25, *p* < 0.012) between tissue contraction and time between procedure and follow-up CT (Fig. 4). In the linear mixed effects model of volume discrepancy, there was a non-zero intercept with effect size (exponentiated coefficients from log(observed/vendor)) of 47.47 (95% confidence interval = 1.94‒1,158.41, *p* = 0.020) consistent with volume discrepancy persisting even after removing tissue contraction, time to follow-up, energy and tumor volume effects (Table 4). Time to follow-up, tumor volume, and ablation energy also had significant effects on the volume discrepancy. In the linear mixed effects model of tip to front edge discrepancy, there was a non-zero intercept with effect size of 206.44 (95% confidence interval = 7.52‒5,666.91, *p* = 0.002), consistent with tip to front edge discrepancy persistent even after removing tissue contraction, time to follow-up, energy, and tumor volume effects (Table 4). Time to follow-up, tissue contraction, and ablation energy also had significant effects on the tip-to-front-edge discrepancy.

**Table 4:** Summary of linear mixed effects model

Discrepancy (observed/vendor)	Fixed effect	Effect size	95% CI	*p*-value
Volume	Intercept	47.47	1.94–1,158.41	0.020
	Time to follow up (per 1 day)	0.97	0.96–0.98	< 0.001
	Tissue contraction (per 1 mm)	0.99	0.93–1.06	0.918
	log(tumor volume)	1.45	1.21–1.73	< 0.001
	log(ablation energy)	0.59	0.41–0.85	0.008
Tip to front edge	Intercept	206.44	7.52–5,666.91	0.002
	Time to follow up (per 1 day)	0.98	0.97–0.99	0.036
	Tissue contraction (per 1 mm)	0.93	0.87–1.00	0.048
	log(tumor volume)	1.12	0.91–1.36	0.273
	log(ablation energy)	0.61	0.41–0.91	0.017

Effect size reflects exponentiated coefficients from log(observed/vendor)

### 3D shape space analysis

**Fig. 5:**
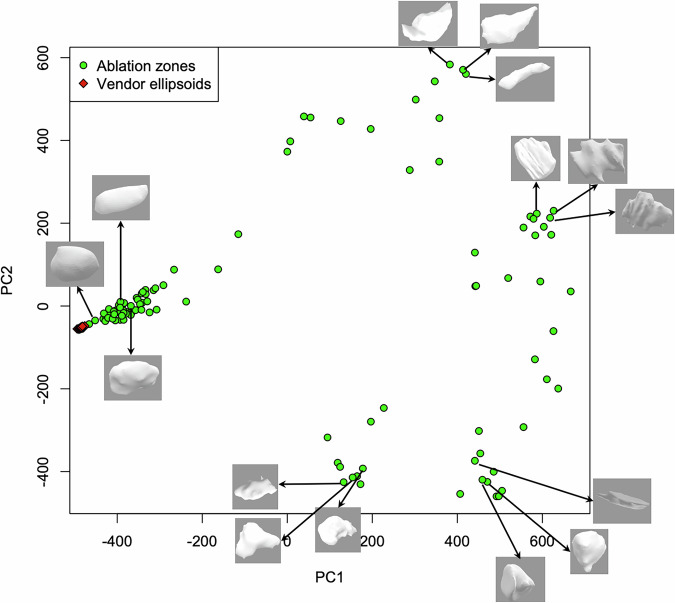
Three-dimensional shape space analysis of ablation zones (green circles) showing the top two principal components. Vendor ellipsoids (red diamonds) all project onto the same location in shape space. Example ablation zones are shown from each grouping in shape space

**Fig. 6:**
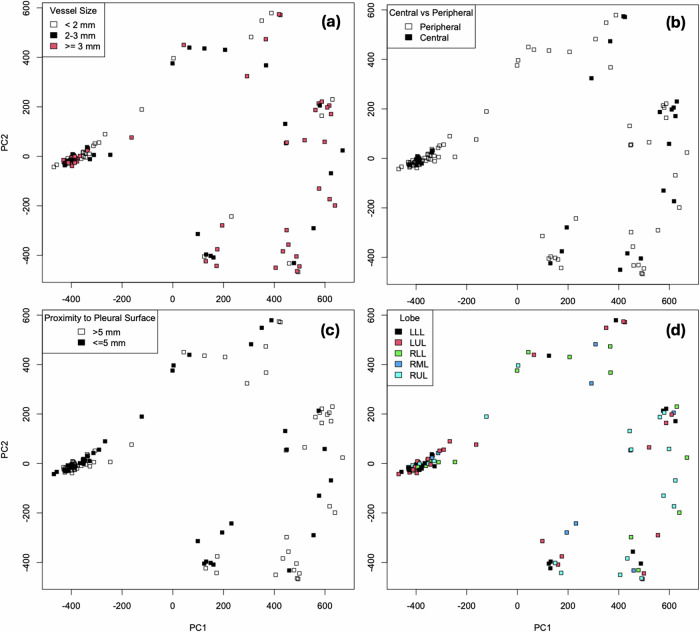
Simple anatomic variables (**a**) vessel size, (**b**) central *versus* peripheral, (**c**) proximity to pleural surface, and (**d**) lobe, do not reproduce shape space groupings observed in shape space

In Fig. 5, each ablation zone (green circle) has been projected onto the top two principal components of the 3D shape space after performing the Euler characteristic transform [17]. Vendor ellipsoids (red diamonds) are projected onto the same space, and all reside in a tight region at the low end of principal component 1. Principal components were not correlated with simple shape descriptors (volume, elongation, sphericity, surface-to-volume ratio). Representative ablation zones are demonstrated in other regions in the shape space. Simple anatomic annotations—lobe, neighboring vessel size, proximity to pleural surface, and central *versus* peripheral location—do not correspond to any location in the shape space (Fig. 6)

## Discussion

LMWA is a well-established technique for treating primary and secondary lung cancers. The current standard for preoperative planning relies on the vendor-provided table of expected ablation zone sizes, but these are based on *ex vivo* animal models. Exploiting an LMWA dataset, this study provides a comprehensive, quantitative analysis of actual lung ablation volumes and shapes. The results demonstrate that while the vendor-provided charts are reasonable estimates of the sample mean volumes, the distribution of ablation volumes is highly variable. Actual ablations extend further past the applicator tip, are longer and wider than predicted by the vendor charts. Ablation zones are asymmetric (the width of the back of the ablation is wider than the width of the front of the ablation) and show striking shape divergence from the vendor model of ellipsoids (Fig. 6). The variability of tissue contraction on the one-month follow-up was not correlated with variability in the ablation zone volume.

Notably, the extent of the variability in the ablation zone volume. There was wide variability for long and short-axis dimensions among ablations with the same power and duration settings, underscoring the limitations of relying on an *ex vivo* animal model (Supplementary Fig. S2). Local anatomy and heat sink effects may modulate at least some of the variability, emphasizing the importance of the operator’s experience in avoiding too small an ablation that could potentially result in local recurrence or too large an ablation that could potentially result in complications. Some authors have suggested that the ablation zone variability is an artifact of post-ablation tissue contraction [8]. Our data does not appear to support this claim. Alternatively, the variability may be largely determined by the local anatomy. While tumor size was correlated with ablation zone volume, tumor imaging characteristics, adjacent vessel size, nodule location, adjacency to pleura, and location between vessels were not associated with volume. Moreover, individually, these simple anatomic covariates did not correlate with ablation zone shape groupings in shape space. It is possible, however, that volume and/or shape are a complex function of these covariates. Notably, a recent manuscript that relies on preoperative CT imaging to train a deep learning model to predict the extent of the ablation zone has shown improvement compared to the existing model [25].

A recent study by Xu et al evaluated microwave lung ablation zone dimensions in a cohort of 31 patients. Similar to our work, they also found ablation zones tended to be longer than the vendor-predicted model [26]. This study relied on long and short-axis dimensions drawn by an expert radiologist, whereas the current study quantified the actual 3D geometric shape variability computed from the segmented ablation zones in addition to comparing *volumes*. The current approach also takes advantage of an ACCS to compare ablations [13]. The current study demonstrated a discrepancy in the distance the ablation zone extends in front of the applicator tip compared to the vendor and asymmetry between the front and the back of the ablation zone. Finally, a formal shape analysis of 3D ablation zones using topological data analysis showed ablation zone shapes that diverge from ellipsoids. We underscore the importance of 3D analysis, which helps to understand how ablation zones can be larger on single-dimension measurements, but have an average volume that is not different from expected. Fundamentally, these ablations are not ellipsoids but rather complex shapes.

The current analysis was performed on the basis of a one-month follow-up CT. While no established standard has been adopted for the timing of the first follow-up scan, recent clinical guidelines have recommended one month as the first baseline scan after lung ablation [11]. Based on this recommendation, the clinical standard at this hospital is to obtain a baseline follow-up CT scan one-month post-ablation. A recently published evaluation of 3D margin assessment after lung ablation also used one month as the standard follow-up [5]. A major potential bias in assessing the ablation at one month is the effect of tissue contraction [27]. In fact, in a recent article evaluating the size of lung ablation zones performed trans-bronchially, tissue contraction was hypothesized as the significant source of variability [8]. Systematic quantification of tissue contraction in the cohort did indeed identify post-ablation variable contraction. However, there was no correlation between tissue contraction and ablation zone volume, power, or duration.

There are several limitations to the current work. First, while our cohort is more extensive than related studies, it is still relatively small. Better estimates of lung ablation zone volume and shape statistics would require a larger cohort that includes more variable ablation parameters, different lung parenchyma backgrounds, and from multiple institutions. Our study focused on only one vendor. However, some of our results are similar to a recent study of a different lung ablation vendor [26], suggesting these observations may be more general to LMWA. To compare with the vendor model, the current study only included LMWA involving one ablation with one applicator. This likely biases the cohort to smaller tumors and smaller ablations. As the focus was on the actual ablation zone statistics of size and shape, the current study did not examine outcomes, including local recurrence rates and complications. We also emphasize that the cohort included only single-device, single-burn, and single-probe cases, limiting generalizability to multi-burn ablations.

In conclusion, this study shows that the vendor-predicted model is a reasonable estimate of the median overall volume of lung ablations. However, there are critically important size and shape distinctions between actual ablations and the vendor-predicted model, which potentially can help clinicians plan treatments. These include the following: (1) the actual ablations are longer than the vendor model; (2) the ablations extend further past the applicator tip but are also narrower in the front than they are in the back, possibly requiring close attention to projected width at the needle tip; (3) the median overall volume can be strikingly variable, underscoring the need for more precise preoperative modeling; and (4) ablation zone shapes span a large space that diverges from ellipsoids. This last point highlights a fundamental problem for practitioners. Prior studies have demonstrated that the minimum margin is associated with local recurrence after ablation [5, 28, 29]. As ablations are not ellipsoids, the margin may well be overestimated if the ablation zone can be narrow in some directions. Further work to understand the relationship between ablation zone shapes and the effect on the margin is warranted.

## Supplementary information

**Additional file 1**: **Fig. S1** Ablation zone width and area, actual (left) and vendor-predicted (right) computed in orientation of the applicator axis. **Fig. S2** Examples of ablation zone size and shape variability for 6 lung ablations performed using the same power of 65 watts and duration of 5 min.

## Data Availability

The datasets and code generated and/or analyzed during the current study are available from the corresponding author on reasonable request and permission from the IRB.

## Abbreviations

3D: Three-dimensional
ACCS: Applicator-centric coordinate system
CT: Computed tomography
IQR: Interquartile range
LMWA: Lung microwave ablation
