# Supplementary material for: Quantitative analysis of lung microwave ablation zone volume and shape
Source: Eur Radiol Exp. 2026 May 28;10:76. doi: 10.1186/s41747-026-00721-2 (PMC13219677; doi:10.1186/s41747-026-00721-2)
Supplement: Supplementary file 1 — Additional file 1: Fig. S1 Ablation zone width and area, actual (left) and vendor-predicted (right) computed in orientation of the applicator axis. Fig. S2 Examples of ablation zone size and shape variability for 6 lung ablations performed using the same power of 65 watts and duration of 5 min. [file 41747_2026_721_MOESM1_ESM.pdf]

Quantitative analysis of lung microwave ablation zone volume and shape

ELECTRONIC SUPPLEMENTARY MATERIAL

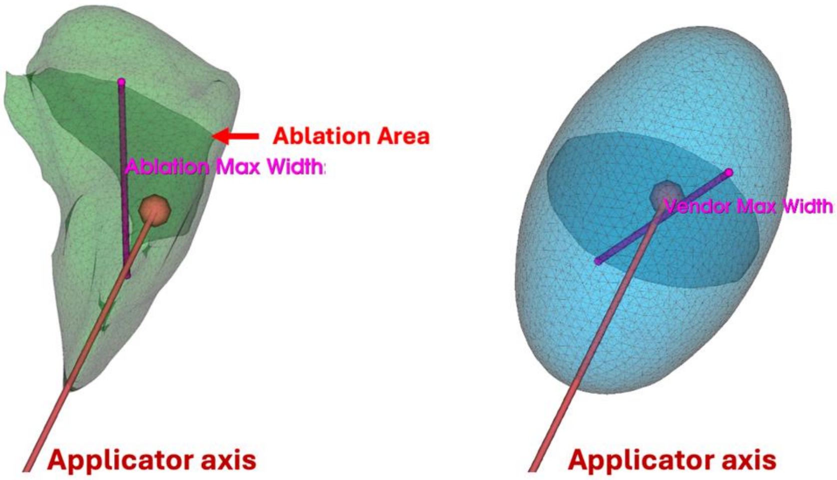

Fig. S1 Ablation zone width and area, actual (left) and vendor-predicted (right) computed in orientation of the applicator axis.

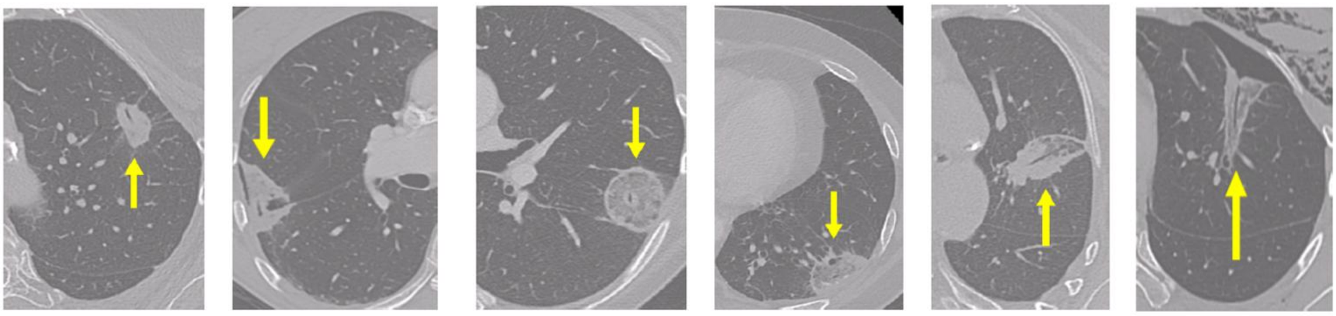

Fig. S2 Examples of ablation zone size and shape variability for 6 lung ablations performed using the same power of 65 watts and duration of 5 min.
